# Hereditary and Sporadic Forms of Aβ-Cerebrovascular Amyloidosis and Relevant Transgenic Mouse Models

**DOI:** 10.3390/ijms10041872

**Published:** 2009-04-23

**Authors:** Samir Kumar-Singh

**Affiliations:** 1 Neurodegenerative Brain Diseases Group, VIB Department of Molecular Genetics, University of Antwerp, Antwerpen - CDE, Universiteitsplein 1, B-2610, Antwerpen, Belgium; E-Mail: samir.kumarsingh@molgen.vib-ua.be; Tel. +3232651002; Fax: +3232651012; 2 Laboratory of Neurogenetics, Institute Born Bunge, University of Antwerp, Antwerpen, Belgium; 3 University of Antwerp, Antwerpen, Belgium

**Keywords:** Alzheimer’s disease, cerebrovascular amyloidosis, CAA, dense-core plaques, senile plaques, amyloid β protein, transgenic mice, pathogenesis, therapy

## Abstract

Cerebral amyloid angiopathy (CAA) refers to the specific deposition of amyloid fibrils in the leptomeningeal and cerebral blood vessel walls, often causing secondary vascular degenerative changes. Although many kinds of peptides are known to be deposited as vascular amyloid, amyloid-β (Aβ)-CAA is the most common type associated with normal aging, sporadic CAA, Alzheimer’s disease (AD) and Down’s syndrome. Moreover, Aβ-CAA is also associated with rare hereditary cerebrovascular amyloidosis due to mutations within the Aβ domain of the amyloid precursor protein (APP) such as Dutch and Flemish APP mutations. Genetics and clinicopathological studies on these familial diseases as well as sporadic conditions have already shown that CAA not only causes haemorrhagic and ischemic strokes, but also leads to progressive dementia. Transgenic mouse models based on familial AD mutations have also successfully reproduced many of the features found in human disease, providing us with important insights into the pathogenesis of CAA. Importantly, such studies have pointed out that specific vastopic Aβ variants or an unaltered Aβ42/Aβ40 ratio favor vascular Aβ deposition over parenchymal plaques, but higher than critical levels of Aβ40 are also observed to be anti-amyloidogenic. These data would be important in the development of therapies targeting amyloid in vessels.

## 1. Introduction

Accumulation of amyloid β-protein (Aβ) in the cerebral vasculature is a common pathological feature of Alzheimer’s disease (AD) and cerebral amyloid angiopathy (CAA) [1,2,3]. Many biochemically distinct types of amyloid have been reported to deposit in cerebral vessels such as Aβ, cystatin-C (Icelandic CAA), transthyretin, gelsolin, prion protein, and the ABri and ADan subunits in familial British and familial Danish dementia [3]. However, CAA caused by Aβ is the most common form of sporadic CAA and also occurs in AD patients [1,2,3].

**Figure 1.**
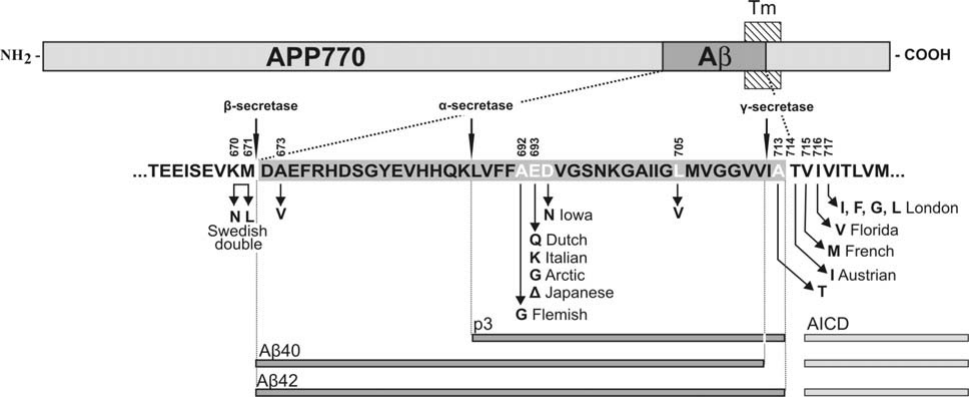
Schematic representation of amyloid precursor protein (APP), positions of the Aβ sequence, transmembrane domain of APP (Tm), and sites of α-, β- and γ-secretase cleavage. NH_2_ and COOH indicate the N-terminus and C-terminus of the protein. Aβ sequence is enlarged below. The constitutive proteolytic cleavage by α- and γ-secretases leads to the formation of the short p3 peptide and processing by β- and γ-secretases leads to Aβ40 and Aβ42 peptides with a consecutive release of a C-terminal APP intracytoplasmic domain (AICD). Also shown are AD/CAA-causing pathogenic amino acid substitutions within APP (see also Table 2 and visit www.molgen.ua.ac.be/ADMutations for a complete and updated list of these mutations).

Aβ is a 40 to 42 aminoacid peptide derived from the amyloid precursor protein (APP), a type 1 transmembrane glycoprotein, through sequential proteolysis by β-secretase and γ-secretase/presenilin complex. A prior cleavage by α-secretase prevents the generation of full-length Aβ and generates p3 (Figure 1). Aβ has a pronounced propensity to form fibrillar aggregates, and the longer, more hydrophobic Aβ42 is also more prone to form fibrils than the shorter Aβ40 isoform. According to the amyloid cascade hypothesis, Aβ deposition is causative for the pathogenesis of AD [4]. One of the most convincing evidence to support this premise is identification of mutations in APP near the β- or γ-secretase cleavage site or in the presenilin gene causing familial forms of AD that increase the absolute or relative levels of Aβ42 [4]. While in these familial forms Aβ is also deposited in vessels, the most severe CAA is observed in patients with APP mutations that lie within the Aβ domain such as Flemish, Dutch, Italian, Arctic, and Iowa, amongst others (Figure 1).

## 2. Clinical and Pathological Consequences of CAA

Aβ CAA (henceforth CAA) is shown to affect leptomeningeal and cortical arteries, arterioles, capillaries, venules and veins in both sporadic and familial CAA, and in almost all AD patients [5,6,1]. Depending on the predominant type of vessel involved, two types of CAA have been distinguished: CAA-type 1 and type 2. CAA-type 1 affects meningeal and cortical arterioles, capillaries, veins and venules while CAA-type 2, also called large-vessel CAA, affects meningeal and cortical vessels but has a tendency to spare cortical capillaries [7]. In CAA-type I, Aβ frequently permeates the glioadventitial junction of capillaries and small arterioles and extends into the parenchyma as “dyshoric angiopathy” [5,8]. Also, depending on the level of vascular involvement, a simple 3-tiered grading system of mild, moderate, and severe CAA has been proposed [9]. In “mild” CAA, focal Aβ deposition restricted to the tunica media occurs in an otherwise normal vessel. In “moderate” CAA, Aβ deposition involves the entire thickness of tunica media, thus completely replacing the smooth muscle cell layer. No evidence of recent or old blood leakage is found. In “severe” CAA, Aβ deposition involves other vascular wall layers and the vascular architecture is severely disrupted with secondary changes such as microaneurysmal dilatation, fibrinoid necrosis, and vessel rupture [9,10,11].

CAA also has important and sometimes fatal pathological consequences. One of the most well-recognized complications of CAA is spontaneous, often recurrent, intracerebral haemorrhage, usually involving the cortex and/or subcortical white matter [12,13,14,9,15,16]. Secondly, CAA also increases the risk of cerebral infarction, especially in the watershed areas, and is frequently associated with ischaemic leucoencephalopathy [17,18,19]. Thirdly, CAA has also been associated with progressive dementia in sporadic CAA patients [20,21,22]. Even in patients devoid of any AD pathology, CAA has been significantly associated with the presence of clinical dementia [23,22]. Interestingly, CAA-type I has been shown to be more closely related to AD pathology than large-vessel CAA [1,24], and increased perivascular phosphorylated tau as well as perivascular inflammation is observed with this type of CAA (see later).

## 3. APP Mutations within the Aβ Sequence Associated with Cerebrovascular Amyloidosis

The genetics of CAA is tightly linked to that of AD. However, research conducted on CAA has been more rewarding in improving our understanding of the biochemistry and molecular genetics of Aβ amyloidogenesis. Not only was Aβ peptide identified first from amyloid deposited in vascular walls in AD and Down’s syndrome patients [25], but the APP mutations were also identified for the first time in a familial form of CAA called hereditary cerebral haemorrhage with amyloidosis – Dutch type (HCHWA-D). HCHWA-D is an autosomal dominant form of CAA characterized by recurrent haemorrhages due to extensive Aβ deposition in cerebral blood vessel walls and diffuse amyloid plaques but absence of dense-core senile plaques or neurofibrillary tau pathology [26,27,28] (Table 1). HCHWA-D patients were identified to carry Dutch APP mutation at codon 693 (APP 770 isoform) replacing a glutamate (E) by a glutamine (Q) [29,30].

**Figure 2.**
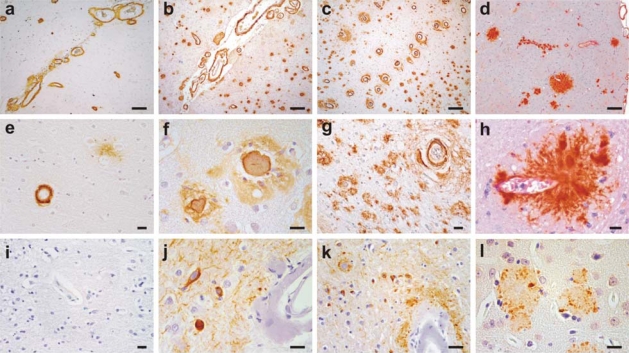
Spectrum of disease from primarily hereditary amyloidosis of Dutch and Flemish type to familial forms of Alzheimer’s disease (AD) and to mouse models of AD. The upper and middle panels depict Aβ staining with monoclonal antibody 4G8 in an HCHWA-D (E693Q) patient with CAA (a, e), a Flemish APP carrier with both CAA and senile plaques (b, f); a presenilin (PS)-1 L282V AD patient with senile plaques and prominent CAA (c, g), and Tg2576 mouse model with parenchymal plaques and CAA (d, h). The lower panel is phosphorylated tau staining (monoclonal antibody AT8) in temporal cortical regions of the same patients (i–k); or ubiquitin staining for Tg2576 (l). Note that HCHWA-D brains lacks dense-core senile plaque or phospho-tau pathology but do show diffuse amyloid plaques (e and i). Flemish APP and PS1 L282V carriers have phosphorylated tau immunostaining in neurofibrillary tangles, ballooned neurites and neuropil threads (j–k). Sections are immunostained with avidin-biotin complex/horseradish peroxidase system and color developed with 3’3’diaminobenzidine. Scale bars in a–d, 200 μm; and e–l, 20 μm.

Soon after, the Flemish APP mutation replacing a glycine (G) by an alanine (A) at the adjacent codon 692 was identified in another Dutch family suffering from presenile dementia and cerebral haemorrhage inherited in an autosomal dominant pattern [31]. Interestingly, clinical phenotypes in Flemish APP carriers overlapped to the extent that cerebral haemorrhages were reported in offspring of patients having dementia while patients with strokes had offspring developing progressive dementia [32,33]. Flemish APP carriers were also shown to have definitive AD with numerous, large senile plaques accompanied by a very severe degree of leptomeningeal and parenchymal CAA [34,33,35]. Moreover, in contrast to HCHWA-D patients, Flemish APP carriers have neurofibrillary tau pathology [33,34] (Figure 2).

Subsequently, other APP mutations within the Aβ sequence were also identified – such as “Italian” (E693K), “Arctic” (E693G), “Japanese” (E693Δ), “Iowa” (D694N) and L705V APP mutations (Table 2; www.molgen.ua.ac.be/ADMutations). The pathology associated with these mutations resembled either Dutch or Flemish APP pathology by having a pure CAA/cerebral haemorrhage or a mixed AD – CAA/cerebral haemorrhage phenotype. For instance, Italian APP and APP L705V mutation resembles the Dutch APP carriers with a predominant CAA with or without recurrent haemorrhages [36,37]. Also, similar to Dutch APP mutation, neurofibrillary tangles (NFT) were absent in APP L705V carriers [37] or were mild and restricted to the archicortex as in the Italian APP carriers [36]. The Arctic and Iowa APP carriers resemble the Flemish APP carriers by having a mixed AD/severe CAA phenotype, although haemorrhagic strokes are not observed [38,39,40,41]. Moreover, consistent to an AD phenotype, parenchymal, ringlike plaques are observed in the Arctic APP carriers [39]. In addition to these APP mutations near the α-secretase site, intragenic Aβ mutations are also reported near the β- and γ-secretase site of APP. For instance, APP A713T mutation near the γ-secretase site reportedly leads to progressive dementia and multiple strokes, and pathological studies confirm AD with severe CAA with multiple infarcts in brain [42]. Similarly, the APP A673V mutation near the β-secretase site of APP has been shown to be associated with AD type of dementia and, consistent with an autosomal recessive pattern of inheritance, is pathogenic only in a homozygous state [43]. Neuropathological data for APP A673V carriers is not yet available [43].

It is clear that APP mutations lying within the Aβ sequence cause pathology by mechanisms distinct from those involving the other APP or presenilin (PS) mutations that cause AD. For instance, the AD-causing mutations in APP or PS were shown to alter APP processing in a manner that increases the absolute or relative levels of Aβ42, the more fibrillogenic Aβ species [47]. Consider, for example, APP Swedish double mutation (K670N/M671L) that leads to increased absolute levels of Aβ42 along with Aβ40 (without changing the Aβ42/Aβ40 ratio), while γ-secretase site APP mutations increase the relative levels of Aβ42 by increasing the ratio of Aβ42/Aβ40 [4]. Similarly, PS1 and PS2 mutations also increased the relative levels of Aβ42 by increasing the ratio Aβ42/Aβ40 [4]. On the other hand, mutations within the Aβ sequence were predicted not only to alter the processing of APP and/or increasing total Aβ production, but were also thought to alter the aggregation properties of the resulting mutant Aβ peptide.

Initial studies seemingly supporting this hypothesis, however, were not completely clear. This was because the Dutch APP peptide showed highly accelerated fibrillogenic kinetics, increased stability, and higher *in vitro* toxicity; while the kinetics of the Flemish Aβ peptide were even slower than the wild-type Aβ and the peptide also did not show any increased toxicity to cells *in vitro* [48,49,50,51]. This obviously contrasted sharply with the clinicopathological consequences as Flemish APP carriers had clinical and pathological AD while the Dutch APP patients although had diffuse type of Aβ deposits, but lacked neuritic, senile plaques characteristic of AD. Subsequent *in vitro* studies, however, showed that compared to wild type and Dutch Aβ, Flemish Aβ was most toxic to differentiated SHSY5Y cells in the early stages of aggregation when Aβ fibrils were not observed [51]. In the late stages of aggregation, the Dutch peptide remained the most toxic Aβ species, suggesting that Aβ, at least under *in vitro* conditions, is neurotoxic in an initial phase due to its soluble oligomeric or other early toxic Aβ intermediate(s), which was distinct from the late neurotoxicity incurred by larger aggregated assemblies of Aβ [51,52]. Moreover, it was also suggested that the cytotoxic potential of Aβ lies in their ability to form extensive fibrils directly on the cell surface, as preaggregation of Aβ abolished its toxic effect on cultured cells [53]. Thus, Dutch Aβ once released from the sites of generation, aggregates almost instantaneously in the parenchymal matrix as non-neuritic diffuse plaques [51,54]. The Flemish APP, on the other hand, not only leads to increased Aβ production due to an increased activity of β-site APP-cleaving enzyme-2 [55], but also slowly fibrillizing mutant Aβ diffuses more efficiently to aggregate as one of the largest dense-core amyloid deposits known in AD. Subsequently, it was also shown that most of the dense-core amyloid plaques observed in the Flemish APP carriers develop in close association with vessel walls [33], perhaps due to entrapment of Aβ in the vascular clearance routes [56,57]. Further observations in Flemish APP carriers showed that CAA-related plaques could also trigger accumulation of tau-immunoreactive dystrophic neurites in the surrounding neuropil similar to dense-core plaques [33] (Figure 2). Vessel association of dense-core plaques might not be unique to Flemish APP carriers as suggested by a recent study showing a significant association between dense-core plaques and Prussian blue-labelled haeme deposits in sporadic AD and Down’s syndrome patients, and even proposing that dense-core plaques are sites of older microhaemorrhages [58].

Subsequent studies on other APP mutations within the Aβ sequence identified similar or additional disease mechanisms. Consider for example, the Italian peptide showing increased propensity to make fibrils and peptide-mediated pathogenic effects similar to the Dutch peptide [59,60]. As a matter of fact, substitutions at APP 693 codon that cause either a loss of charge (E22Q; Dutch APP) or a change of charge (E22K; Italian APP) show increased binding to and degeneration of cerebrovascular smooth muscle cells [59] and, not surprisingly, both of them lead to CAA/haemorrhagic strokes. On the other hand, complete deletion of APP 693 codon as in Japanese APP shows altered aggregation property of enhanced oligomerization but no fibrillization [45]. This is similar to the Arctic APP variant that also increases the propensity of protofibril formation without increasing the rate of fibril formation or the production of Aβ [38]. And lastly, the Iowa APP carriers that resemble Flemish and Dutch APP carriers show cytotoxicity and aggregation properties that lie between those caused by the Flemish and Dutch Aβ peptides [60]. Interestingly, Flemish, Dutch, Italian, Arctic, and Japanese Aβ variants are also shown to be more resistant to proteolytic degradation [61,45,62]. Recent data also suggest that Dutch, Italian, and Iowa preferably assemble in the presence of GM3 ganglioside [63] while their clearance across the blood-brain barrier (BBB) might be reduced as shown for Dutch/Iowa mutant [64,65]. It remains to be shown that oligomers of the variant Aβ that are associated with AD changes are more synaptotoxic than wild type Aβ oligomers [45].

## 4. Other Genetic Risk Factors for CAA

Copy number variations of the APP gene also seem to be important for development of CAA. For instance, Down’s syndrome patients who have three copies of APP, have CAA as young as 30 years and the severity of CAA increases with age [66]. Down’s syndrome patients are also reported to have strokes [67,68,69]. Similarly, duplications of the APP locus in certain French and Dutch families or populations demonstrate an autosomal dominant AD and/or lobar cerebral haemorrhage with prominent CAA [70,71]. APP duplications increase the absolute amounts of both Aβ42 and Aβ40 (without changing the ratio), an important factor proposed to lead to Aβ deposition in vessel walls [33,72]. Although uncommon, a predominant CAA is also observed in select PS1 mutations like Q184D and L282V, amongst others [73,74,75,76,77]. The best studied PS2 mutation, N141I, has also been reported to lead to haemorrhagic strokes in Volga-German family [78]. It remains to be shown whether some of these mutations, especially those occurring after codon 200 [79], might not alter Aβ40 production as has been shown for the majority of PS mutations [80,81].

Specific apolipoprotein E (APOE) alleles are not only a strong risk factor for development of AD, but are also linked to development of CAA and strokes. CAA related to capillaries and smaller arterioles is observed to be closely associated with ApoE-ε4, but not ApoE-ε2 [7]. ApoE-ε4 is also independently associated with increased vascular Aβ deposition in large-vessel CAA leading to strokes [82,83,84]. On the other hand, ApoE-ε2 appears to promote degenerative changes in the amyloid-laden vessel wall and is a cause of stroke independent of Aβ deposition [85,86]. Anecdotal examples of individuals homozygous for the APOE-ε4 allele that have a predominant CAA or a CAA-related pathology are also described [23]. Lastly, candidate genes involving Aβ degradation pathways have also been implicated in the development of CAA [87]. For instance, a polymorphism in the Neprilysin gene has been shown to be associated with CAA [88]. For many of these candidate genes, knockout mouse models have been made as discussed in the next section.

## 5. Mouse Models of Cerebrovascular Amyloidosis

Most disease models are based on inherited forms of disease that although less common than the sporadic forms, are clinically indistinguishable from each other. The first attempts to model the archetypal Dutch and Flemish APP cerebral amyloidosis in mice did not show amyloid till at least 18 months of age [89,90], however, recent experiments have successfully created Dutch APP mice [91]. At ≈ 25 months of age, APP Dutch mice demonstrate extensive CAA, smooth muscle cell degeneration and haemorrhages – features that are typical of HCHWA-D [91]. Similarly, transgenic mice expressing Dutch/Iowa mutant Aβ (Tg-SwDI) at levels below those of endogenous mouse APP have also been established that robustly deposit Aβ, particularly in the cerebral microvasculature, from 3 months of age [92,93]. However, because mutant Aβ is expected to have altered biophysical properties, any direct extrapolation to sporadic CAA has to be made with caution.

**Figure 3.**
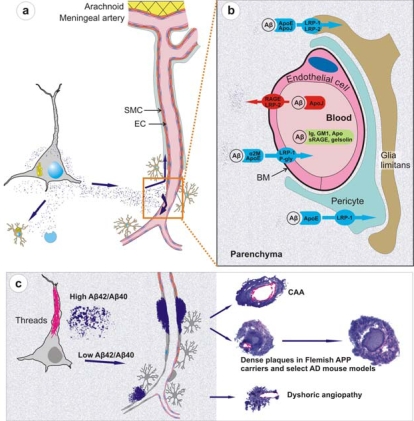
Physiological clearance and pathological deposition of Aβ in brain. (a) The newly synthesized Aβ is locally degraded by glial uptake and cell-associated and extracellular proteases or cleared along the periarterial interstitial fluid pathway or directly across the BBB through specific receptors or carrier-mediated mechanisms (EC, endothelial cell; SMC, smooth muscle cell). (b) At the level of BBB, Aβ is transported into the blood flow via a transcytosis mechanism mediated by LRP-1 and P glycoprotein transporters, in association with α2 macroglobulin and ApoE. A reverse transport from the blood towards the parenchyma is mediated by RAGE and LRP-2 receptors in association with apolipoprotein J. In addition, Aβ might be sequestered in the blood flow by immunoglobulins, ganglioside GM1, apolipoproteins, soluble RAGE receptors and gelsolin. (c) Astrocyte endfeet and pericytes also mediate Aβ intake by expressing LRP-1 and -2 receptors. As the production of Aβ exceeds its clearance, Aβ starts to deposit. It is possible that in situations of high Aβ42/Aβ40 ratio, highly fibrillogenic Aβ42 deposits near the site of production as diffuse plaques. It is also likely that such plaques sequester newly synthesized Aβ and mature to dense plaques. However, in situations where Aβ42/Aβ40 ratio remains unaltered or low, the major Aβ gradient is set towards vessels where Aβ42 seeds the deposition of more abundantly produced, and more diffusible, Aβ40 to form vessel-related CAA and dense plaques. Figure is not drawn to scale (adapted from Pirici *et al.*, with permission from the publisher [118].

Interestingly, mice overexpressing wild type Aβ such as APP Swedish mice (APP/Sw; e.g., Tg2576 and APP23 mice), or APP London mice, develop CAA fairly early and have been used as a valid model for CAA [94,95,96](www.alzforum.org/res/com/tra). Importantly, APP/Sw mice with unaltered relative ratios of Aβ42/Aβ40 are even more representative of sporadic CAA/AD and have been employed in several studies. In principle, these mouse models have provided support for many important concepts in our understanding of cerebral amyloidosis including Aβ deposition in vessel walls, as summarized below:

1. Mouse models have given a definitive view that Aβ42 is essential for Aβ deposition not only in parenchyma but also in vessels. When APP/Sw mice (producing both Aβ40 and Aβ42) are bred with mice expressing mutant PS1 (that increases brain Aβ42/Aβ40 ratio), the crossbred mice have drastically increased amyloid deposition in parenchyma and vessels compared to single transgenic controls [97,98]. These APP/PS1 mice also have a higher number but smaller sizes of amyloid deposits most likely due to the extra “seeds” provided by Aβ42 [99]. A similar crossbreeding of APP Dutch mice with mice expressing AD-related PS1 G384A mutation also increases amyloid depositions and shifts the pathology from vascular to the parenchymal compartment [91]. The premise that Aβ42 is essential for Aβ deposition is also neatly answered by BRI-Aβ40 and BRI-Aβ42 mice that produce only Aβ40 or Aβ42 [100]. In these mice, a fusion construct is utilized wherein the carboxyl terminus sequence of BRI protein, involved in amyloid deposition in familial British and Danish dementia, is replaced by a sequence encoding either Aβ40 or Aβ42. A proteolytic cleavage of this fusion protein at a furin cleavage site immediately preceding Aβ results in high-levels of Aβ40 or Aβ42 secretion. While BRI-Aβ40 mice expressing high levels of Aβ40 do not develop overt amyloid pathology, the BRI-Aβ42 mice line expressing lower levels of Aβ42 develop all types of brain amyloid deposits including CAA [100]. Crossbreeding of BRI-Aβ42 mice with Tg2576 mice again leads to a massive increase in amyloid deposition. These data establish that Aβ42 is essential for amyloid deposition in the parenchyma and also in vessels.
2. Mouse models have also supported a “protective” role of Aβ40 in plaque deposition and therefore in AD pathology, especially when the levels of Aβ40 exceed a critical level. The initial data to support a protective role of Aβ40 came from *in vitro* studies where Aβ40 was shown to directly interfere with Aβ42 aggregation by delaying the Aβ42-mediated nucleation step at an early stage in the fibrillogenesis process [101]. More recently, γ-secretase site APP mutations, like the Austrian (T714I) and French (V715M) mutations, have also been shown to cause a drastic decrease in Aβ40 production [102,103]. For instance, Austrian APP reduces Aβ40 by ≈ 80% and because Aβ40 is the major physiologically produced peptide (≈ 9 times more than Aβ42), a sharp reduction in total Aβ also occurs [102,104]. A similar decrease in absolute Aβ40 levels has also been shown for a number of clinical PS mutants [80,81] and interestingly, age-of-onset of PS1-linked AD not only correlates inversely with Aβ42 but also directly with Aβ40 levels [80,105]. Further studies on mouse models have also provided compelling data to support the premise that Aβ40 is anti-amyloidotic. First, results from transgenic mice expressing wild-type and various mutant forms of APP suggest that increased Aβ40 levels reduce amyloid deposition [106]. Secondly, at least two independent studies utilizing knockin PS familial AD mutations crossbred with Tg2576 mice show a greatly accelerated plaque pathology accompanied by decreased production of Aβ40 without an increase in secreted brain Aβ42 levels [107,108]. Similarly, BRI-Aβ40 mice crossbred with either BRI-Aβ42 or Tg2576 mice show greatly reduced brain amyloid deposition compared to singly transgenic BRI-Aβ42 or Tg2576 mice [100]. These data all suggest that in the absence of Aβ42, the clearance of Aβ40 is very high, and depending upon the critical levels of Aβ42, Aβ40 might even be anti-amyloidotic [72].
3. Recent work on knockout and transgenic mice has provided evidence that Aβ is cleared from brain by mechanisms involving microglial uptake, degradation by cellular or extracellular proteases, as well as by clearance through vascular route. For instance, *in vivo* imaging of live mice has shown that Aβ is not only taken up by microglia but astrocytes also play a direct role in degradation of Aβ [109]. Secondly, knockout mouse models have supported data of Aβ degradation by brain proteases such as neprilysin, insulin-degrading enzyme (IDE), endothelin-converting enzymes-1 and -2, and matrix metalloproteinase-2 and -9 [110]. Consider, for example, neprilysin homozygous knockout mice expressing mutant APP and showing an expectedly higher burden of brain amyloid including CAA [111]. Thirdly, studies on mouse models have demonstrated that Aβ that cannot be locally degraded has the potential to diffuse away from the site of production. Non-transgenic brain tissue grafted in APP23 hosts develop both diffuse and congophilic amyloid plaques [112]. And finally, experimental studies in mice have shown that diffusible Aβ is transported directly across the BBB into the blood, mediated by low density lipoprotein receptor-related protein-1 (LRP-1)/α2-macroglobulin and ApoE [113,57]. Mouse models of mutant Aβ have also shown that some of these mutant Aβ such as the Dutch/Iowa mutants could be deficient in its clearance across the BBB explaining the robust CAA seen in these mutation carriers (Deane *et al*., 2004; Davis *et al*., 2006). Studies on rodents also support a second vessel-related Aβ clearance route along the periarterial spaces [56,113,114]. Exogenous tracer studies in rats show that tracers injected into the parenchyma of grey matter in the rat brain drain along perivascular spaces around leptomeningeal arteries to the base of the brain, pass through the cribriform plate and, via nasal lymphatics, to deep cervical lymph nodes [115].
4. Studies on mouse models have indicated that local production of Aβ by vascular elements is not absolutely essential for the development of CAA. This is important as, for instance, hypoxia followed by reoxygenation in microvascular smooth vessel cells has been shown to upregulate APP and proposed to be an initiating event in the pathogenesis of amyloid angiopathy [116]. However, mice that solely produce APP/Aβ from neuronal cells in an endogenous APP knockout background drive Aβ pathology in both parenchyma and vessels [114]. Furthermore, these studies also lend support to the hypothesis that failure of Aβ vascular drainage and entrapment of Aβ in the periarterial space leads to development of CAA [56]. Similarly, pathological studies on some of the mouse models have suggested that in certain situations, dense plaques in mouse models are also associated with vascular walls as has been proposed earlier for sporadic AD [117] and shown for Flemish APP pathology [33]. Recent data from Tg2576 and PSAPP mice show that up to 90% of these dense plaques, but not diffuse plaques, are centred on vessel walls or reside in the immediate perivascular regions [99]. Similar observations have also been made on Tg-SwDI mice where all dense plaques were observed to be associated with vessel walls [93]. These data suggest that the mechanisms involved in the formation of “neuritic” dense (core) plaques in select AD such as Flemish APP pathology or in select transgenic mice such as Tg2576 and PSAPP mice are similar to CAA formation, but distinct from those involved in the formation of non-neuritic, diffuse plaques (Figure 3).
5. Mouse models demonstrate that similar to large vessel-CAA in humans [119], amyloid associated with vessel walls is predominantly of the Aβ40 type. A similar biochemical profile is also observed for the compact dense-core plaques, which is in contrast to diffuse plaques that are predominantly composed of Aβ42 [120]. Conversely, situations that lead to a drastic reduction of Aβ40 also drastically reduce the prevalence of CAA and dense-core plaques as observed in Austrian APP pathology [102]. Tg2576 and PSAPP mouse models preferentially producing Aβ40 also deposit Aβ40-enriched cerebrovascular amyloid [121,99]. Dutch APP mice also deposit more mutant Aβ40 than mutant Aβ42 in vessels [91]. Furthermore, experimental work on rodents has demonstrated that Aβ40 is more prone to compact as dense deposits in contrast to the faster aggregating Aβ42 that preferentially deposits as diffuse plaques. When soluble Aβ40 and Aβ42 are injected in rat brain, soluble Aβ40 forms congophilic, fibrillar dense deposits while Aβ1-42 forms only diffuse deposits [122]. Lastly, BRI-Aβ42 mice that solely secrete Aβ42 also have Aβ42 peptide trafficked and deposited in vessels [100]. Thus, mouse models support the viewpoint that although “Aβ40” plays an important role in development of vessel-associated compact plaques, most likely due to a more efficient vascular clearance and its high abundance, Aβ42 on its own also has the potential to migrate and deposit in association with vessels; and without a critical relative level of Aβ42, Aβ40 clearance is too efficient to allow deposition.
6. Mouse models have partly elucidated the role of ApoE which is otherwise poorly understood. ApoE has been shown to bind to Aβ [123], and studies on transgenic mice suggest that ApoE, especially ApoE-ε4, has a role in Aβ fibrillization [124]. When APP London mice are crossbred with ApoE knockout mice, Aβ chiefly deposits as diffuse, nonfibrillar plaques. However, when these mice were crossbred with mice transgenically expressing human ApoE-ε4, they develop far more fibrillogenic, dense-core plaques and CAA than when crossbred with mice expressing the ApoE-ε3 isoform [124]. Because dense-core plaques and CAA are rich in Aβ40, these data suggest that ApoE-ε4 has a role in Aβ40 fibrillization as also suggested by studies on AD [125].
7. Transgenic mouse models serve as a useful model to study CAA-associated pathological changes. Vascular Aβ deposits in mouse models are shown to cause degeneration of vascular smooth muscle cells and of other vascular components typically identified in AD and hereditary cerebral amyloidosis [126,91,11]. Some mouse models also show ultrastructural microvascular abnormalities in non-amyloidotic vessels such as endothelial cell loss, basement membrane thickening, and degeneration of smooth muscle cells and pericytes as shown for AD [99,11]. Additionally, mouse models have shown that basement membrane abnormalities could also contribute to development of CAA as capillary basement membrane thickening precedes the development of CAA in TGF-β transgenic mice [127]. And lastly, transgenic mouse models such as APP23 and Tg2576 have shown that vascular amyloidosis is indeed the cause of spontaneous haemorrhages as both cerebral microhaemorrhages and fatal lobar haemorrhages occur in these mice [126,99].
8. Finally, mouse models of amyloidosis have proved to be essential in testing therapeutic amyloid targeting from vessels. A number of active and passive immunotherapeutic approaches such as peripheral sequestering utilizing non-immune mechanisms have been successfully tried in these mouse models [128,129,130,131,132]. In an active immunization trial on an AD mouse model, behavioural and cognitive abnormalities were shown to be reversed coinciding with ≈ 50% reduction in dense-core plaques [129]. Similarly, a passive immunization approach in Tg2576 mouse model has been shown to revert some of the BBB abnormalities observed in these mice [133]. Furthermore, mouse models also reproduce some of the side effects of anti-Aβ vaccinations. For instance, similar to one of the encephalitic patients from the Aβ active immunization trial revealing presence of multiple cortical haemorrhages in association with Tcell inflammatory infiltrates [134], APP23 mice receiving passive anti-Aβ immunization were also shown to have infrequent but severe CAA-associated microhaemorrhages [135]. Furthermore, similar to breakdown of BBB seen in Tg2576 [99], autopsy of one of the encephalitic patients from the active immunization trial revealed that antibody titers in cerebrospinal fluid equalled those in plasma, again indicating a severe breakdown of the BBB [136]. These data indicate that mouse models of amyloidosis could be instrumental in understanding some of the ill effects of anti-amyloid drug targeting. However, data from mouse models should always be viewed with caution as mouse models also have serious limitations as reviewed recently [72]. As an example, vaccination trials targeting Aβ N-terminus in humanized mouse models could easily miss the adverse effects caused by sequestration of physiological Aβ because human and murine Aβ have a different N-terminus. Despite these limitations, mouse models would continue to provide important clues in the understanding of the processes involved in vascular amyloidosis and in causing dementia.

## 6. Conclusions

In this review, I have discussed how molecular pathology of hereditary cerebrovascular amyloidosis due to APP mutations within the Aβ domain, as well as experimental studies in transgenic mouse models have helped to partially dissect molecular mechanisms involved in Aβ cerebrovascular amyloidosis. Firstly, the mutant forms of Aβ causing AD/cerebral haemorrhage were discussed that strongly support the role of APP in the disease mechanism and provide one of the strongest rationales for studies of factors that influence abnormal metabolism and aggregation of Aβ in the causation of CAA with or without AD. Secondly, studies on hereditary cerebrovascular amyloidosis and mouse models were discussed that suggest that while an absolute or relative increase in Aβ42 or an increased Aβ42/Aβ40 ratio is important for parenchymal plaque deposition and development of an AD phenotype, increased Aβ with an unaltered Aβ42/Aβ40 ratio favours cerebrovascular amyloidosis. However, critical relative levels of Aβ42 are also important as without this, the clearance of Aβ is too efficient to allow deposition. Thirdly, these data suggest that a low fibrillogenic potential of Aβ could also favour plaque compaction as is the case with Flemish Aβ that leads to the formation of large, neuritic, dense-core plaques. And lastly, by illuminating the relationships between specific lesions such as CAA and dense-core plaques and their molecular components, studies on hereditary cerebrovascular amyloidosis and mouse models have shown that CAA and dense-core plaques could be a spectrum of the same disease mechanism. Thus, molecular pathological studies of cerebrovascular amyloidosis and relevant mouse models would continue to provide detailed insights into the pathogenesis of CAA and contribute to the development of targeted therapeutic strategies.

## Figures and Tables

**Table 1.** Extracellular cerebral amyloid deposits commonly present in familial and sporadic Aβ-CAA and AD patients and transgenic mouse models.

CAA	Cerebral amyloid angiopathy; pathological changes occurring in cerebral blood vessels caused by deposition of amyloid protein of different origins, but here due to Aβ.
Dense-core plaques	Also referred to as “neuritic”, “senile”, “classic”, or “mature” plaques. The central compact dense-core is surrounded by a corona of diffuse plaque. The corona contains predominantly Aβ42 and is ThS-negative. The core is ThS-positive. Almost always associated with proximate phospho-tau pathology.
Dense plaques	ThS-positive compact amyloid without the corona of diffuse plaques. Major compact plaque type in transgenic mouse models. Biochemically resembles cores of the dense-core plaques. In this review, also sometimes used to include dense-core plaques.
Diffuse plaques	Loosely arranged fibrils that are usually ThS-negative. Best recognized with immunohistochemistry and are constituted predominantly of Aβ42. Are not commonly associated with proximate phospho-tau pathology.

**Table 2.** Clinicopathological spectrum for APP mutations within Aβ1-43 coding sequence.

APP Codon*	Substitution	Position within Aβ	Proximity to secretases	Nickname	Disease	References
673	Ala→Val	A2V	β-secretase			[43]

692	Ala→Gly	A21G	α-secretase	Flemish	AD/ Cerebral haemorrhage	[31,33]
		
693	Glu→Gln	E22Q	merge with α-secretase	Dutch	Cerebral haemorrhage	[29,44]
	Glu→Lys	E22K		Italian	Cerebral haemorrhage	[36]
		
693 (merge the cell with above)	Glu→Gly	E22G	also α- secretase (merge the cell with above)	Arctic	AD/ severe CAA but no strokes	[38,39]
	Glu–>Del	E22Δ				
				Japanese	AD; pathology unavailable	[45]
694	Asp→Asn	D23N				
				Iowa	AD/ severe CAA but no strokes	[40,41]
705	Leu→Val	L34V				
				-	Severe CAA	[37]

713	Ala→Thr	A42T	γ-secretase	-	AD/ Cerebral haemorrhage	[42]
		
714	Thr→Iso	T43I	also γ- secretase(merge with above)	Austrian	AD	[46]

* Numbered according to the largest APP transcript APP770.
